# GSDMD and GSDME exhibit distinct roles in enteric coronavirus PDCoV-induced pyroptosis and inflammatory responses

**DOI:** 10.1128/jvi.01876-24

**Published:** 2025-06-12

**Authors:** Chenyu Li, Yuting Shi, Chunying Xie, Kaiqi Duan, Tong Ding, Xiangfei Xu, Liurong Fang, Yanrong Zhou, Shaobo Xiao

**Affiliations:** 1 Key Laboratory of Preventive Veterinary Medicine in Hubei Province, Cooperative Innovation Center for Sustainable Pig Production, Wuhan, China; 2 National Key Laboratory of Agricultural Microbiology, College of Veterinary Medicine, Huazhong Agriculture University 47895, Wuhan, China; Loyola University Chicago - Health Sciences Campus, Maywood, Illinois, USA

**Keywords:** porcine deltacoronavirus (PDCoV), 3C-like protease (3CL^pro^), gasdermin D, gasdermin E, inflammatory response

## Abstract

Porcine deltacoronavirus (PDCoV), an emerging enteric coronavirus with zoonotic potential, typically causes intestinal villous epithelial cell damage with inflammation. Pyroptosis is a recently identified inflammatory form of programmed cell death that has been found to be associated with the pathogenesis of many viruses. However, the effects of PDCoV infection on pyroptosis and the role of pyroptosis in its pathogenesis remain unclear. In this study, we report that PDCoV infection triggers pyroptosis, as demonstrated in porcine ileum epithelial cell lines and intestinal tissues of PDCoV-infected piglets. Although both gasdermin D (GSDMD)- and gasdermin E (GSDME)-mediated pyroptosis were observed during PDCoV infection, GSDME dominated PDCoV-induced pyroptosis and subsequent inflammatory responses. More differently, GSDMD, rather than GSDME, exhibited potent anti-PDCoV activity; however, PDCoV-encoded nonstructural protein 5, a 3C-like protease, cleaved GSDMD, but not GSDME, to abolish the antiviral and pyroptotic functions of GSDMD. Our study elucidates the distinct roles of GSDMD and GSDME in PDCoV-induced pyroptosis and inflammatory responses, providing new insight into the pathogenesis of PDCoV and the potential for anti-PDCoV drug development.

**IMPORTANCE:**

Pyroptosis is a type of programmed cell death mediated by various gasdermins (GSDMs). While previous research has primarily focused on the role of GSDMD in pyroptosis, our study demonstrates that GSDME plays a dominant role in pyroptosis and the concomitant inflammatory responses induced by porcine deltacoronavirus (PDCoV), a newly identified enteric coronavirus with the potential to infect humans. The cleavage of GSDMD by PDCoV 3C-like protease may account for the diminished functionality of GSDMD in PDCoV-induced pyroptosis, which simultaneously disrupts its antiviral potential against PDCoV. These findings reveal the intricate interplay between PDCoV, GSDMD, and GSDME, accelerating the elucidation of PDCoV pathogenicity.

## INTRODUCTION

Porcine deltacoronavirus (PDCoV) is a single-stranded, positive-sense, enveloped RNA virus that belongs to the *Deltacoronavirus* genus within the *Coronaviridae* family of the *Nidovirales* order (1, 2). Since its initial detection in Hong Kong in 2012, outbreaks of PDCoV in industrialized pig farms have been reported globally (2, 3). PDCoV is currently considered one of the critical causes of viral diarrhea in piglets, mainly leading to vomiting, acute diarrhea, dehydration, and even mortality in newborn piglets. Pathologically, PDCoV infection primarily results in thinning and hyalinization of the intestinal wall, as well as atrophy of intestinal villi, along with intestinal inflammation (4). In addition to the gut, PDCoV can be detected in other tissues such as the heart, spleen, and kidney, indicating its broad tissue tropism (4, 5). Importantly, several studies have demonstrated that PDCoV can infect not only pigs but also calves, chickens, turkey poults, and mice (6–10). Recently, three cases of PDCoV infection in Haitian children with acute undifferentiated febrile illness were reported, highlighting the potential for cross-species transmission and zoonotic spread of PDCoV (11). Therefore, it is crucial to elucidate the pathogenesis of PDCoV.

Pyroptosis is a recently discovered form of programmed cell death (12, 13). During pyroptosis, cells exhibit specific characteristics, including cell swelling, pore formation, and the release of cytoplasmic contents (14, 15). In 2015, Shao and colleagues identified the gasdermin (GSDM) family members as essential molecules for triggering pyroptosis (13). The GSDM family comprises GSDMA, GSDMB, GSDMC, GSDMD, GSDME, and pejvakin (PJVK) (13). The GSDMs, except for PJVK, share a similar structure consisting of a highly conserved and cytotoxic N-terminal domain, a repressor C-terminal domain, and a hinge loop connecting the two domains (13). The cleavage of the hinge loop connecting the N-terminal and C-terminal domains of GSDMs (excluding PJVK) by some proteases, particularly caspases, is a prerequisite for GSDMs to induce GSDMD-mediated pyroptosis (13, 16–18). For example, caspase-1/4/5/8/11 are known to cleave GSDMD, which results in GSDMD-mediated pyroptosis, and under pathogenic infection, caspase-1 cleavage of GSDMD is the most common occurrence of GSDMD-mediated pyroptosis (13, 19). Moreover, caspase-3 is capable of processing GSDME to mediate GSDME-mediated pyroptosis, and it also has the ability to cleave GSDMD, which ultimately results in the inability of GSDMD-mediated pyroptosis to occur (16, 20).

Pyroptosis has been shown to be associated with the infection and pathogenicity of many microorganisms. In addition to the identified inhibitory effects of GSDM-mediated pyroptosis on the proliferation of viruses or bacteria (21–27), a growing number of studies have reported that pyroptosis is usually accompanied by robust inflammatory responses, which in turn cause severe tissue damage. Previously, most studies focused on the association between GSDMD-mediated pyroptosis and inflammatory responses (13, 16–18). For example, infections with pseudorabies virus, severe acute respiratory syndrome coronavirus 2 (SARS-CoV-2), respiratory syncytial virus, influenza A virus (IAV), and *Streptococcus* induce GSDMD-mediated pyroptosis to elevate the release of proinflammatory cytokines (22, 28–34). Moreover, several studies have demonstrated that GSDME-mediated pyroptosis can occur in certain circumstances of viral infection (35–40). Furthermore, certain conditions of viral, such as SARS-CoV-2, human coronavirus 229E (HCoV-229E), and enterovirus 71 (EV71) infection, have been observed to induce both GSDMD- and GSDME-mediated pyroptosis (25, 35, 37, 40, 41). It is noteworthy that a recent study on Zika virus (ZIKV) noted that GSDME knockout mice exhibited less severe pathological changes and a significantly lower incidence of abnormal pregnancies and adverse fetuses compared to wild-type (WT) mice (37). Moreover, another study showed that H7N9 IAV infection can trigger GSDME-mediated pyroptosis, thereby causing a lethal cytokine storm and lung injury (36). This highlights the necessity of investigating the potential roles of GSDMs, especially GSDME, in the induction of inflammatory responses and subsequent pathological injury by viral infections.

In this study, we demonstrated that PDCoV infection triggers pyroptosis mediated by both GSDMD and GSDME. GSDMD-induced pyroptosis suppresses PDCoV replication, while GSDME-mediated pyroptosis plays a more predominant role in PDCoV-induced inflammatory responses. However, PDCoV-encoded nonstructural protein 5 (nsp5), a 3C-like protease (3CL^pro^), cleaves GSDMD to counteract its antiviral effect. These findings provide novel insights into understanding PDCoV pathogenesis.

## RESULTS

### PDCoV infection triggers lytic cell death *in vitro* and *in vivo*

**Fig 1:**
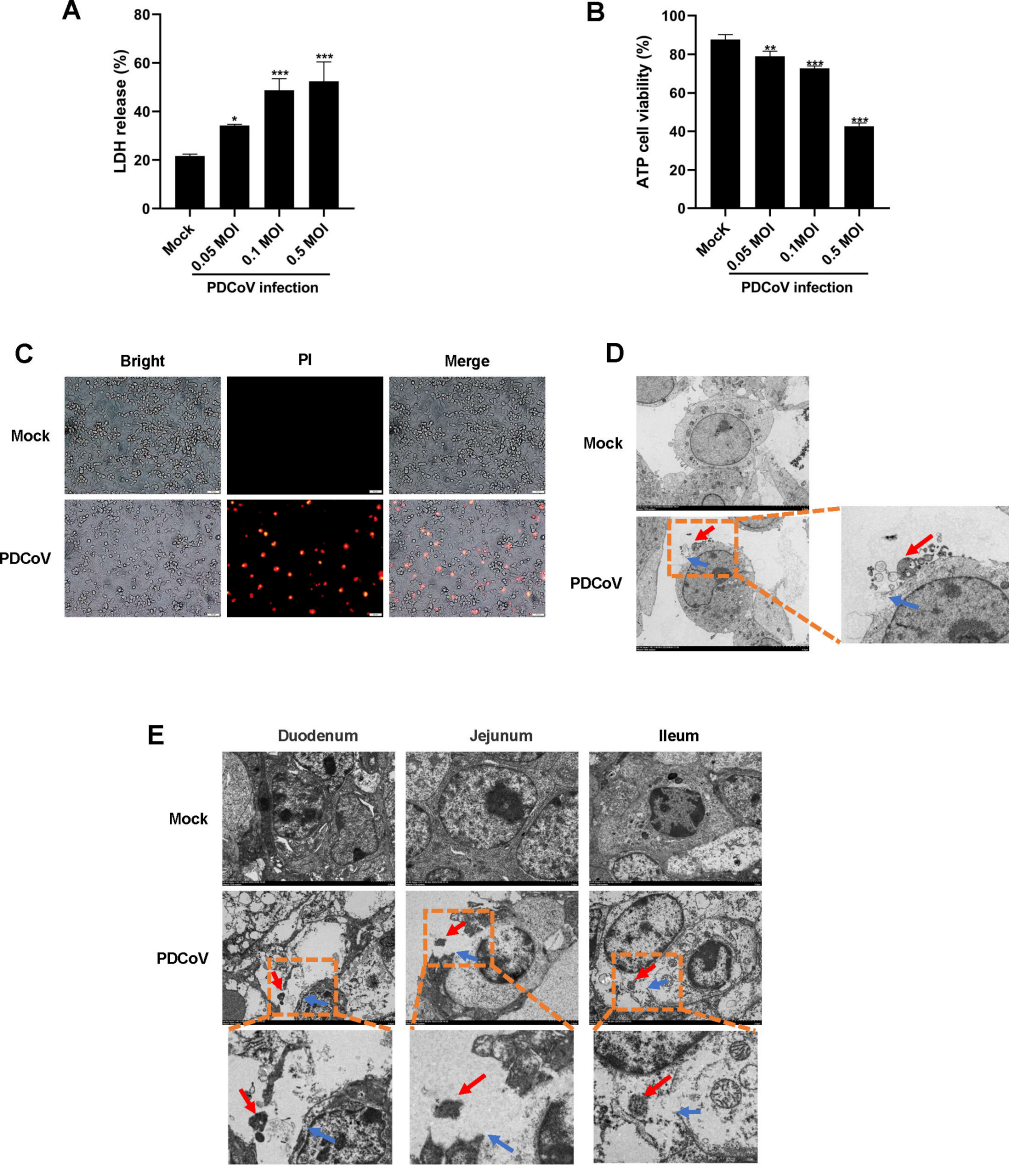
PDCoV infection induces lytic cell death. (**A and B**) IPI-2I cells were infected with PDCoV (0.05, 0.1, and 0.5 multiplicity of infection [MOI]). At 24 h post-infection, LDH release (**A**) and ATP cell viability (**B**) were measured. Values are shown as the mean ± SD from three independent experiments. *, *P* < 0.05; **, *P* < 0.01; ***, *P* < 0.001. (**C**) PI staining of IPI-2I cells infected or mock-infected with PDCoV (0.5 MOI) for 24 h. (**D**) Morphological changes of IPI-2I cells after infection with PDCoV for 24 h were observed by transmission electron microscopy. (**E**) Cellular morphological changes of porcine intestinal tissues after infection with PDCoV were observed by transmission electron microscopy.

PDCoV infection in nursing piglets results in swelling and vacuolization of intestinal epithelial cells (4). Thus, we speculated that PDCoV infection might induce lytic cell death. To test this conjecture, we assessed the impact of PDCoV infection on lytic cell death using IPI-2I cells, a line of porcine ileum epithelial cells that are the primary target cells of PDCoV infection *in vivo*. Lactate dehydrogenase (LDH) release (an indicator of lytic cell death) and cell viability assessed by adenosine triphosphate production were measured. The results showed that PDCoV infection significantly promoted LDH release (Fig. 1A) and decreased cell viability (Fig. 1B). To further determine the occurrence of lytic cell death, we employed the red fluorescent dye propidium iodide (PI) to explore whether PDCoV infection causes cell membrane rupture. As shown in Fig. 1C, red fluorescence was observed in PDCoV-infected cells, while no obvious red fluorescence signal was detected in mock-infected cells. Additionally, transmission electron microscopy (TEM) revealed distinct morphological features of lytic cell death in PDCoV-infected cells, including cell membrane rupture (blue arrows) and the release of cytoplasmic contents (red arrows), whereas the morphology of mock-infected cells did not undergo obvious changes (Fig. 1D). Taken together, these findings indicate that PDCoV infection triggers lytic cell death *in vitro*.

We further investigated whether PDCoV infection induces lytic cell death *in vivo*. In the intestinal tissues of piglets, we observed cell membrane rupture (blue arrows) and the release of cytoplasmic contents (red arrows) in the intestinal epithelial cells of PDCoV-infected piglets, but not in the mock-infected piglets (Fig. 1E; Fig. S1). These results demonstrate that lytic cell death occurs during PDCoV infection *in vivo*.

### PDCoV infection promotes GSDMD- and GSDME-mediated pyroptosis

**Fig 2:**
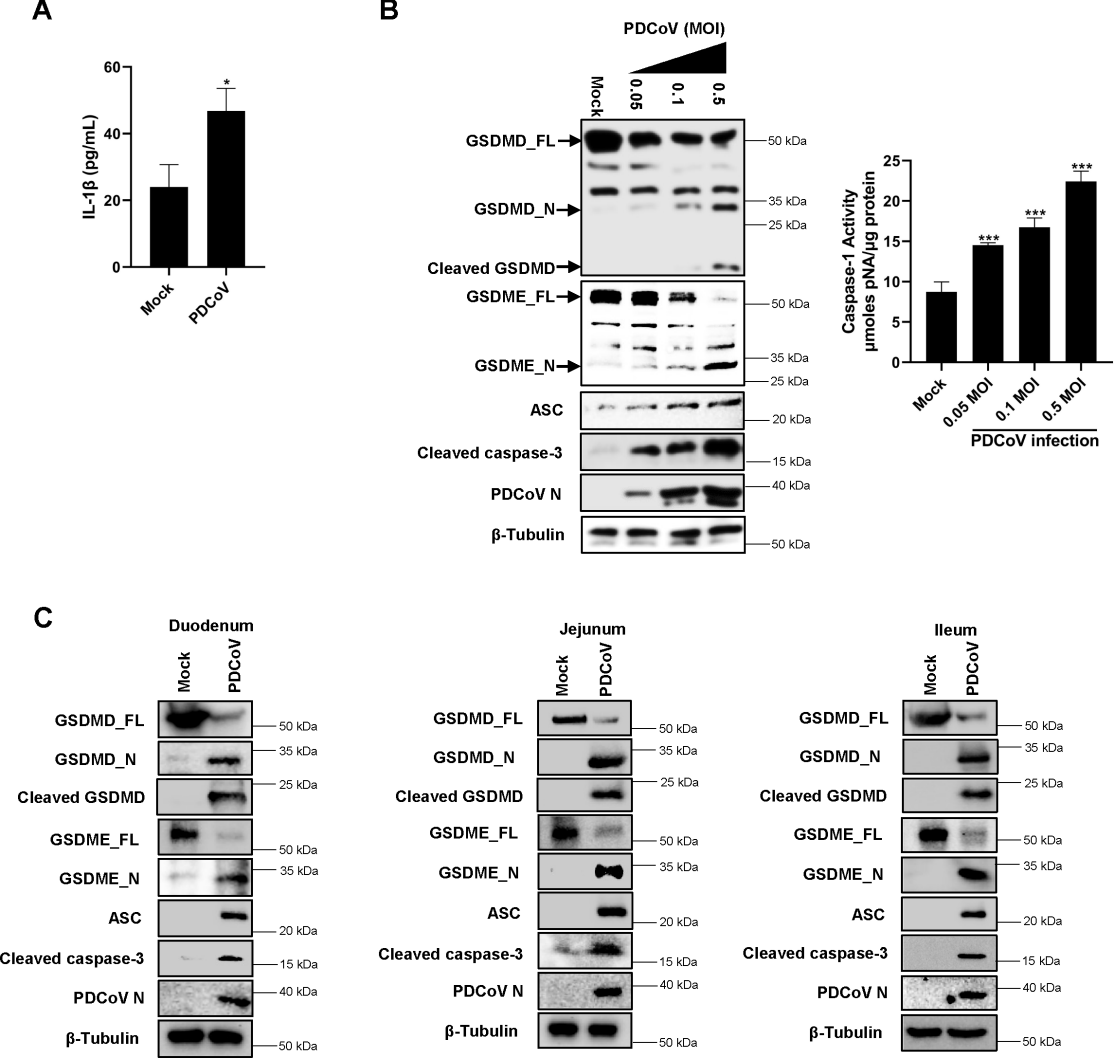
PDCoV infection promotes GSDMD- and GSDME-mediated pyroptosis. (**A**) IPI-2I cells were infected with PDCoV (0.5 MOI). Cell supernatants were collected at 24 h post-infection, and the protein levels of IL-1β were assessed using ELISA. Values are shown as the mean ± SD from three independent experiments. *, *P* < 0.05. (**B**) IPI-2I cells were infected with PDCoV (0.05, 0.1, and 0.5 MOI). At 24 h post-infection, cell lysates were harvested for analysis. β-Tubulin was used as a loading control in the western blot analysis. The activity of activated caspase-1 was quantified using a Caspase-1 Activity Assay Kit, following the manufacturer’s instructions. Values are shown as the mean ± SD from three independent experiments. ***, *P* < 0.001. (**C**) Protein expression levels of GSDMD, GSDME, ASC, and caspase-3 in the intestinal tissues of piglets mock-infected or infected with PDCoV were determined by western blot analysis. β-Tubulin was used as a loading control.

Given that PDCoV infection is frequently accompanied by inflammation and that pyroptosis is a highly inflammatory form of lytic programmed cell death (4, 42), we speculated that PDCoV infection may induce pyroptosis. To test this speculation, we evaluated the effect of PDCoV infection on the secretion of interleukin-1β (IL-1β), a widely recognized biomarker for pyroptosis. The results indicated that the protein levels of IL-1β in the cell supernatants were significantly elevated following PDCoV infection (Fig. 2A), suggesting the occurrence of pyroptosis.

Previous studies have demonstrated that pyroptosis primarily occurs through the activation of GSDMD and GSDME (13, 16). Therefore, we further examined the effects of PDCoV infection on GSDMD and GSDME. Our results revealed that PDCoV infection caused dose-dependent decreases in full-length GSDMD and full-length GSDME (hereafter referred to as GSDMD_FL and GSDME_FL, respectively), as well as increases in GSDMD N-terminal and GSDME N-terminal (hereafter referred to as GSDMD_N and GSDME_N, respectively) in IPI-2I cells (Fig. 2B). Similar findings were observed in the intestinal tissues of piglets infected with PDCoV (Fig. 2C).

Previous studies have shown that GSDMD and GSMDE are primarily cleaved by activated caspase-1 and caspase-3 (16, 43, 44). To investigate how PDCoV infection mediates the cleavage of GSDMD and GSDME, we examined the activity changes of caspase-1 and caspase-3 following PDCoV infection. Western blot analysis revealed that the protein levels of cleaved caspase-3 and ASC were upregulated by PDCoV infection both *in vitro* and *in vivo* (Fig. 2B and C). Additionally, using a Caspase-1 Activity Assay Kit, we found that PDCoV infection dose-dependently increased caspase-1 activity (Fig. 2B). Notably, although GSDMD has been reported to be proteolytically processed by both caspase-1 and caspase-3, generating a 32 kDa fragment (denoted GSDMD_N) and a 43 kDa fragment, respectively (43, 44), we found that the 43 kDa fragment was undetectable in PDCoV-infected cells via western blot analysis. This finding suggests that caspase-3 may not be involved in PDCoV-induced GSDMD cleavage in pigs, or that its capacity to cleave GSDMD is substantially lower compared to that of caspase-1. Consequently, we propose that PDCoV infection activates both the caspase-1–GSDMD axis and the caspase-3–GSDME axis.

### GSDME dominates PDCoV infection-induced pyroptosis

**Fig 3:**
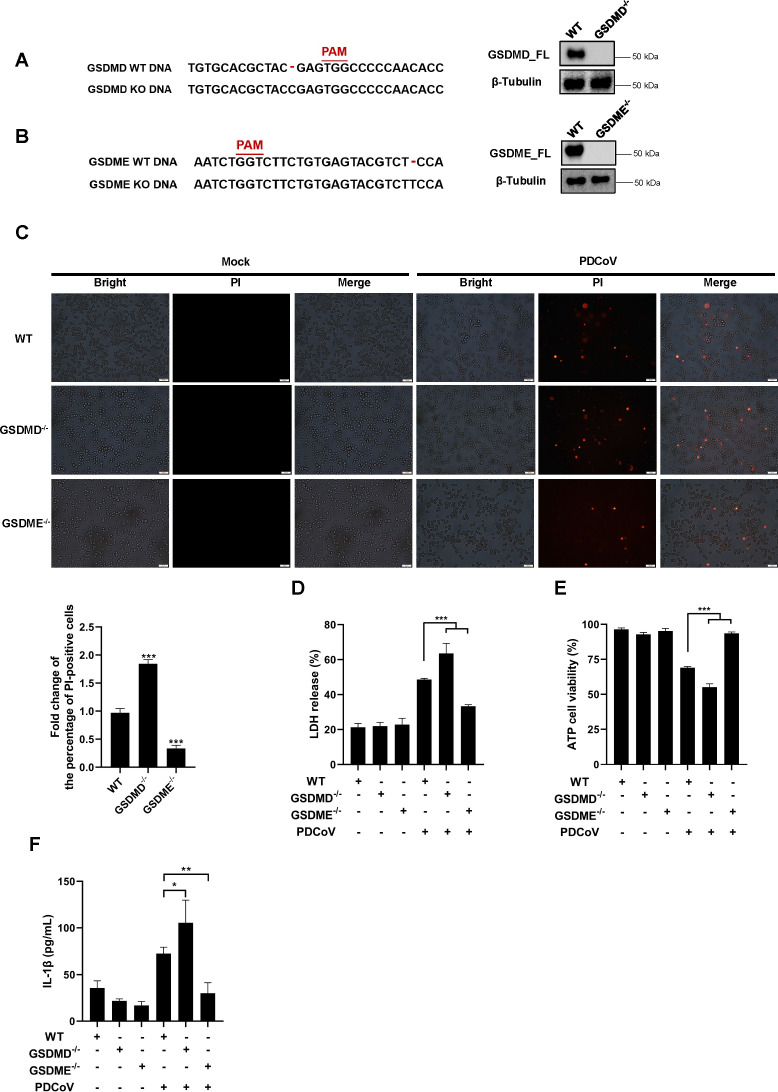
GSDME plays a dominant role in PDCoV infection-induced inflammatory responses. (**A and B**) The successful construction of GSDMD^−/−^ (**A**) and GSDME^−/−^ (**B**) IPI-2I cell lines was verified via Sanger sequencing and western blot analysis. β-Tubulin was used as a loading control. (C–F) WT, GSDMD^−/−^, and GSDME^−/−^ IPI-2I cells were infected or mock-infected with PDCoV (0.5 MOI) for 24 h. Subsequently, PI staining (**C**), LDH release (**D**), ATP cell viability (**E**), and the protein levels of IL-1β (**F**) were measured. The percentage of PI-positive cells in (**C**) was quantified using ImageJ software. Cell supernatants were collected for the detection of IL-1β protein levels using ELISA (**F**). In (D–F), values are shown as the mean ± SD from three independent experiments. *, *P* < 0.05; **, *P* < 0.01; and ***, *P* < 0.001.

To further investigate the contribution of GSDMD and GSDME to the induction of pyroptosis by PDCoV infection, we constructed GSDMD-knockout (GSDMD^−/−^) and GSDME-knockout (GSDME^−/−^) IPI-2I cell lines (Fig. 3A and B). PI staining assays showed that upon PDCoV infection, the number of PI-positive cells increased in the GSDMD^−/−^ cells and decreased in the GSDME^−/−^ cells compared with the corresponding WT cells (Fig. 3C). These findings suggest that GSDME knockout downregulated the levels of pyroptosis, whereas GSDMD knockout upregulated pyroptosis levels during PDCoV infection. To further confirm this conclusion, we assessed LDH release, cell viability, and IL-1β secretion in WT, GSDME^−/−^, and GSDMD^−/−^ IPI-2I cell lines. The results revealed that GSDME^−/−^ cells showed significant decreases in LDH release and IL-1β secretion, as well as a notable increase in cell viability compared with WT cells during PDCoV infection, whereas GSDMD^−/−^ cells exhibited the opposite trend (Fig. 3D through F). These results align with the results of PI staining assays. Altogether, these findings indicate that GSDME, rather than GSDMD, is the primary molecule responsible for pyroptosis induction during PDCoV infection.

### PDCoV infection-induced inflammatory responses are primarily mediated by GSDME rather than GSDMD

**Fig 4:**
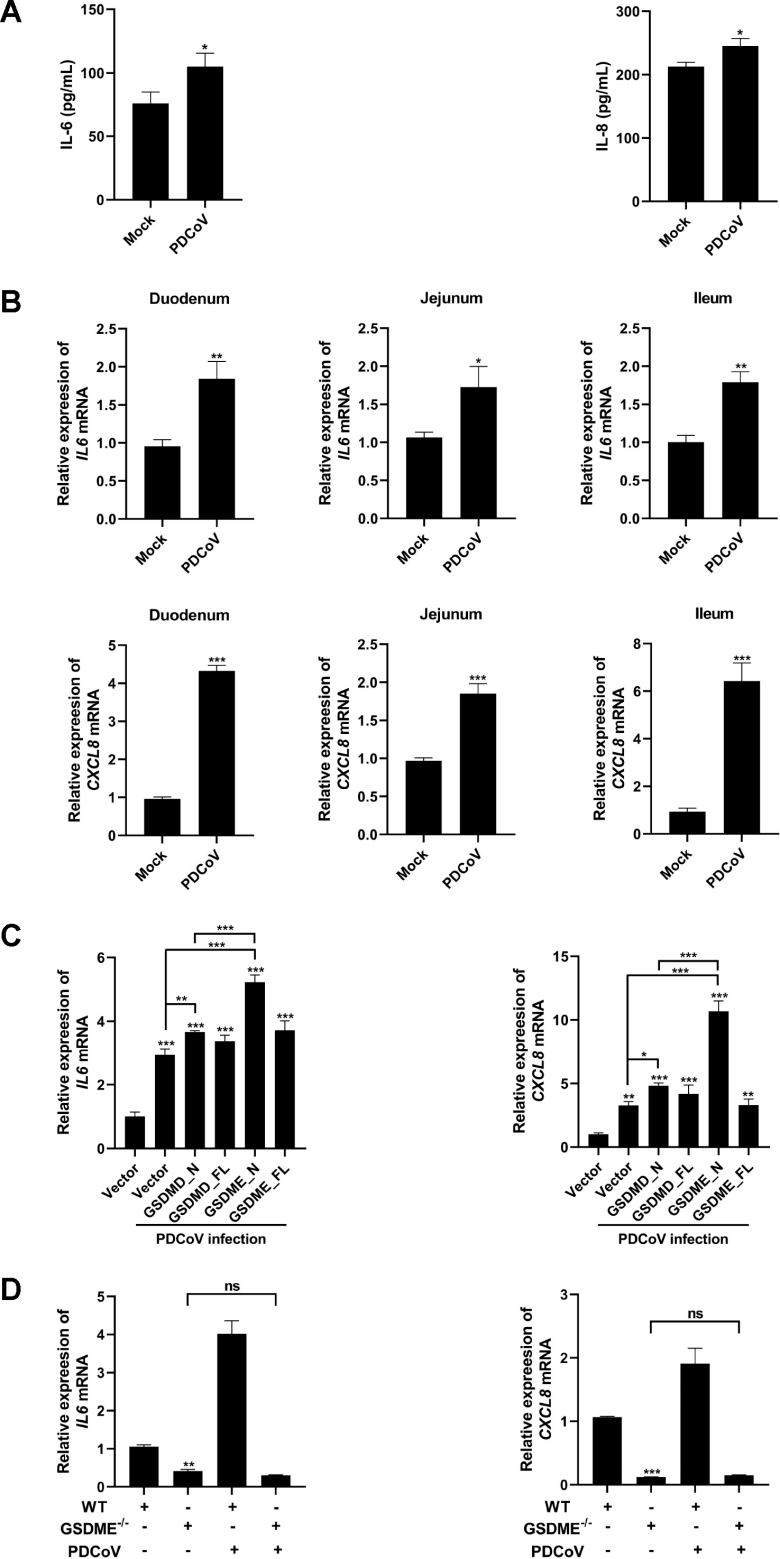
GSDME is a more important inducer of inflammatory responses than GSDMD upon PDCoV infection. (**A**) IPI-2I cells were infected or mock-infected with PDCoV (0.5 MOI) for 24 h. Cell supernatants were collected, and the protein levels of IL-6 and IL-8 were assessed using ELISA. (B–D) The relative mRNA levels of *IL6* and *CXCL8* were analyzed by qRT-PCR. *ACTB* was used as the internal control. (**B**) Intestinal tissues in piglets inoculated or mock-inoculated with PDCoV were collected for qRT-PCR. (**C**) IPI-2I cells were transfected with eukaryotic expression plasmids encoding GSDMD_FL, GSDME_FL, GSDMD_N, and GSDME_N for 24 h, followed by infection with PDCoV (0.5 MOI) for an additional 24 h. (**D**) WT and GSDME^-/-^ IPI-2I cells were infected or mock-infected with PDCoV (0.5 MOI) for 24 h. Values are shown as the mean ± SD from three independent experiments. *, *P* < 0.05; **, *P* < 0.01; and ***, *P* < 0.001; ns, not significant.

Pyroptosis is considered a form of inflammatory cell death due to its frequent occurrence alongside an inflammatory response (13, 16). Therefore, we further explored whether PDCoV infection elicits inflammatory responses. The results revealed significant upregulations of IL-6 and IL-8 protein levels in cell supernatants, as well as *IL6* and *CXCL8* mRNA levels, in PDCoV-infected IPI-2I cells compared with mock-infected cells (Fig. 4A; Fig. S2). Similarly, the mRNA levels of *IL6* and *CXCL8* in the intestinal tissues of PDCoV-infected piglets were also significantly upregulated (Fig. 4B). These results suggest that PDCoV infection induces inflammatory responses both *in vitro* and *in vivo*.

We further compared the effects of GSDMD and GSDME on the inflammatory response induced by PDCoV infection. To this end, IPI-2I cells were transfected with eukaryotic expression plasmids encoding GSDMD_FL, GSDME_FL, GSDMD_N, or GSDME_N, followed by PDCoV infection. The results showed that both GSDMD_N and GSDME_N significantly upregulated the mRNA levels of *IL6* and *CXCL8*, with GSDME_N exhibiting a greater ability to upregulate these proinflammatory cytokines than GSDMD_N (Fig. 4C), suggesting that GSDME induces more intense inflammatory responses than GSDMD. To further confirm the role of GSDME in the inflammatory responses induced by PDCoV infection, GSDME^−/−^ IPI-2I cell lines were used here. The data revealed that GSDME knockout resulted in significant decreases in the mRNA levels of proinflammatory cytokines (*IL6* and *CXCL8*) (Fig. 4D). In particular, PDCoV-induced upregulation of proinflammatory cytokine levels in GSDME^−/−^ cells was almost completely abolished (Fig. 4D). Taken together, these findings indicate that GSDME, rather than GSDMD, plays a predominant role in the induction of inflammatory responses by PDCoV infection.

### Cleavage of GSDMD by PDCoV nsp5 diminishes its promotion of inflammatory responses

**Fig 5:**
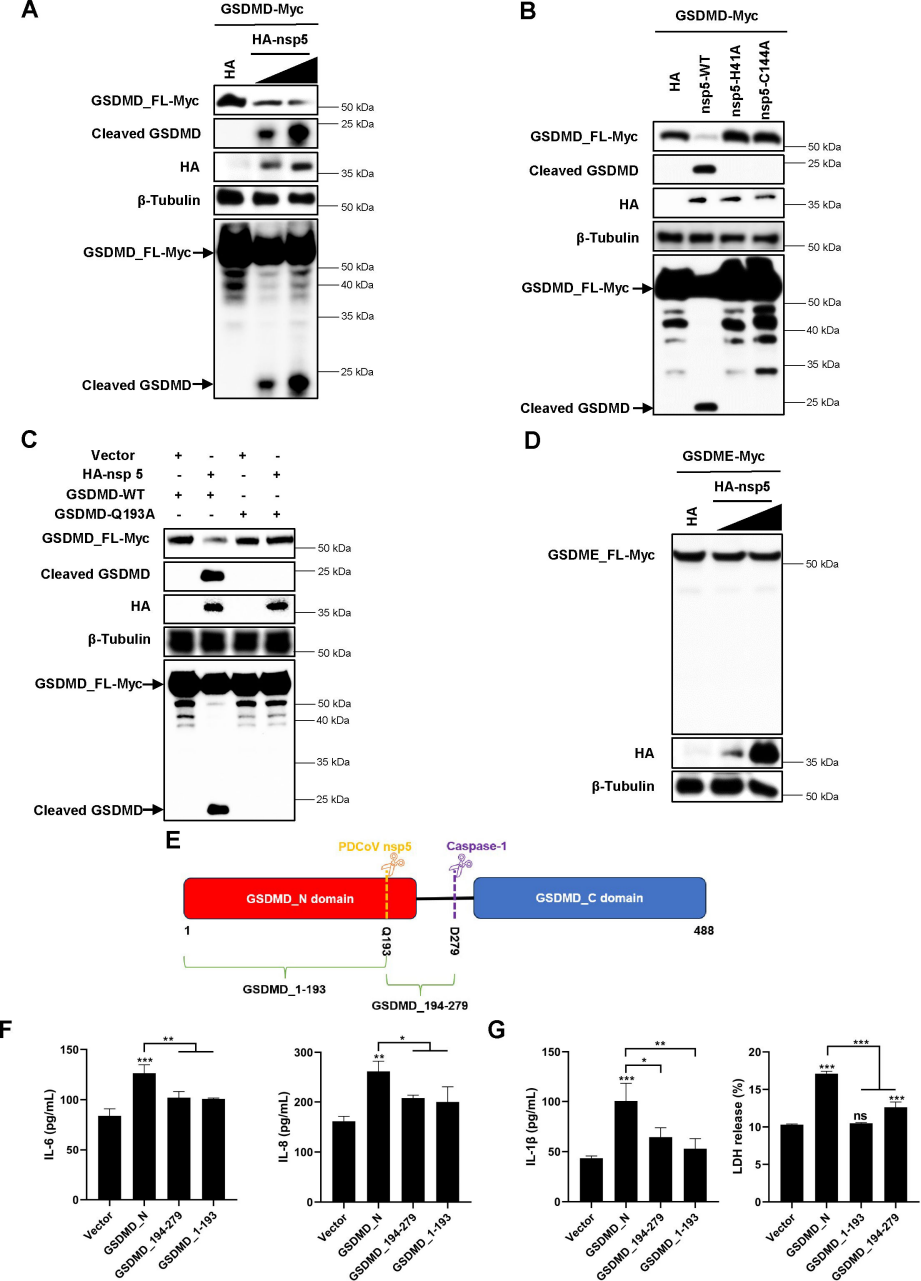
PDCoV nsp5 cleaves GSDMD but not GSDME to impair the proinflammatory activity of GSDMD. (A–E) Expression levels of GSDMD_FL and cleaved GSDMD were determined by western blot analysis. β-Tubulin was used as a loading control. (**A**) HEK-293T cells were cotransfected with eukaryotic expression plasmids encoding Myc-tagged GSDMD_FL and HA-tagged PDCoV nsp5. After 24 h, cells were lysed for immunoblotting. (**B**) HEK-293T cells were cotransfected with eukaryotic expression plasmids encoding Myc-tagged GSDMD_FL and HA-tagged PDCoV nsp5 (wild-type) or nsp5 protease-defective mutants (H41A and C144A). After 24 h, cells were lysed for immunoblotting. (**C**) HEK-293T cells were cotransfected with eukaryotic expression plasmids encoding HA-tagged PDCoV nsp5 and Myc-tagged GSDMD_FL (wild-type GSDMD_FL, abbreviated as GSDMD-WT) or GSDMD_FL mutant (GSDMD-Q193A). Cells were lysed after 24 h and evaluated by immunoblotting. (**D**) HEK-293T cells were cotransfected with eukaryotic expression plasmids encoding Myc-tagged GSDME_FL and HA-tagged PDCoV nsp5. After 24 h, cells were lysed for immunoblotting. (**E**) Schematic representation of GSDMD and its cleavage fragments induced by caspase-1 or PDCoV nsp5. (**F and G**) IPI-2I cells were transfected with plasmids encoding GSDMD_N, GSDMD_1-193, and GSDMD_194-279. Cell supernatants were collected at 24 h after transfection. The protein levels of IL-6 (**F**), IL-8 (**F**), and IL-1β (**G**) were assessed using ELISA. LDH release was measured using CytoTox 96 Non-Radioactive Cytotoxicity Assay Kit (**G**). In (**F and G**), values are shown as the mean ± SD from three independent experiments. *, *P* < 0.05; **, *P* < 0.01; and ***, *P* < 0.001; ns, not significant.

Next, we aimed to investigate the underlying mechanisms restricting the contribution of GSDMD to the inflammatory responses induced by PDCoV infection. A previous study reported that PDCoV cleaves GSDMD at glutamine (Q) 193 to produce the GSDMD_1-193 and GSDMD_194-279 fragments through viral nsp5, also known as 3CL^pro^ (26). Consistently, in the present study, we observed that nsp5 cleaved GSDMD, resulting in the generation of a ~23 kDa band (Fig. 5A). The cleavage of GSDMD was also detected in PDCoV-infected IPI-2I cells and the intestinal tissues of PDCoV-infected piglets (Fig. 2B and C). Moreover, this cleavage was found to be dependent on the 3CL^pro^ activity of nsp5 (Fig. 5B) and occurred at the Q193 site of GSDMD (Fig. 5C). However, PDCoV nsp5 did not exhibit cleavage activity on GSDME (Fig. 5D). Collectively, these findings indicate that PDCoV nsp5 cleaves GSDMD, but not GSDME, both *in vitro* and *in vivo*. On this basis, we hypothesized that the cleavage of GSDMD by PDCoV nsp5 might limit the role of GSDMD in triggering inflammatory responses during PDCoV infection.

Considering that GSDMD_N is responsible for GSDMD-induced inflammatory responses (Fig. 4C) and PDCoV nsp5-mediated cleavage of GSDMD_N generates smaller fragments including GSDMD_1-193 and GSDMD_194-279 (Fig. 5E), we next compared the impact of these fragments on inflammatory responses with GSDMD_N. The results showed that GSDMD_1-193 and GSDMD_194-279 induced significantly lower protein levels of IL-6 and IL-8 in cell supernatants, as well as mRNA levels of *IL6* and *CXCL8*, compared with GSDMD_N (Fig. 5F; Fig. S3). These findings suggest that PDCoV nsp5 cleaves GSDMD_N, resulting in its limited contribution to inflammatory responses during PDCoV infection. Notably, we also observed that nsp5-mediated cleavage of GSDMD_N diminished its pyroptotic function (Fig. 5G), providing further evidence for the possible association between the proinflammatory and pyroptotic properties of GSDMD.

### PDCoV nsp5 cleaves GSDMD to abolish its antiviral activity

**Fig 6:**
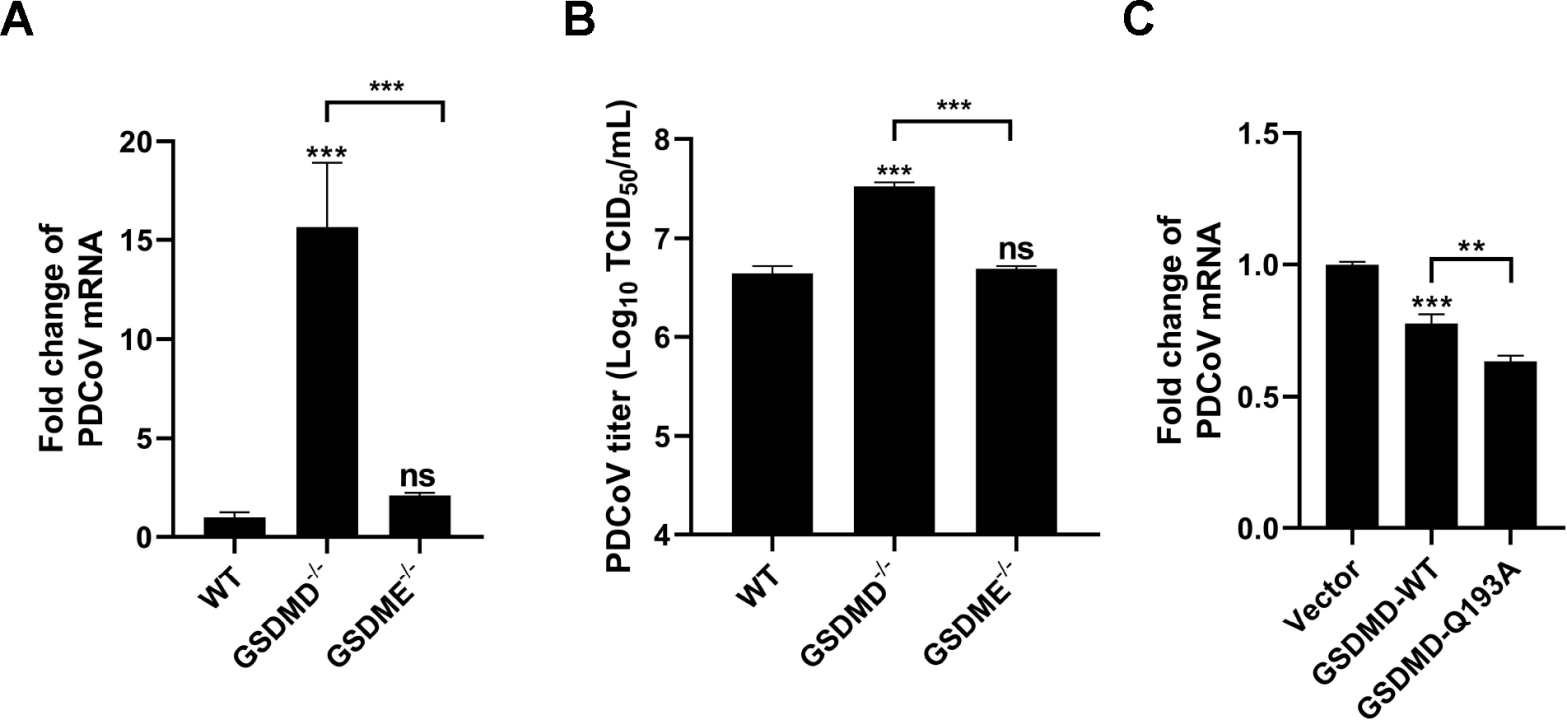
Cleavage of GSDMD_ N by PDCoV nsp5 abolishes its antiviral activity. (**A and B**) WT, GSDMD^−/−^, and GSDME^−/−^ IPI-2I cells were infected with PDCoV at an MOI of 0.5 for 24 h. The PDCoV RNA copy number (**A**) and viral titer (**B**) were measured. (**C**) GSDMD^−/−^ IPI-2I cells were transfected with eukaryotic expression plasmids encoding GSDMD-WT or GSDMD-Q193A. At 24 h after transfection, cells were infected with PDCoV at an MOI of 0.5. At 24 h post-infection, the RNA copy number of PDCoV was measured. Values are shown as the mean ± SD from three independent experiments. **, *P* < 0.01; ***, *P* < 0.001; ns, not significant.

We further explored why PDCoV nsp5 cleaves GSDMD but not GSDME. A previous study demonstrated the role of GSDMD as an antagonist against PDCoV infection using a swine testicular (ST) cell line (21). Herein, using GSDMD^−/−^ IPI-2I cell lines, we also observed that knockout of GSDMD significantly increased both the mRNA levels and titers of PDCoV, demonstrating the anti-PDCoV effect of GSDMD (Fig. 6A and B; Fig. S4). However, different from GSDMD, GSDME knockout did not have a significant impact on PDCoV multiplication at all tested time points (12, 18, and 24 h post-infection) (Fig. 6A and B; Fig. S4). We therefore speculated that PDCoV nsp5 cleaves GSDMD to block its antiviral activity. To test this speculation, we compared the effects of wild-type GSDMD (GSDMD-WT) with a cleavage-resistant GSDMD mutant (GSDMD-Q193A), in which the Q193 site was mutated to A193, rendering it unable to be cleaved by PDCoV nsp5, on PDCoV replication. As shown in Fig. 6C, the GSDMD-Q193A mutant exhibited significantly higher anti-PDCoV activity in comparison to GSDMD-WT (Fig. 6C). Altogether, these findings indicate that PDCoV nsp5 cleaves GSDMD to benefit viral proliferation.

## DISCUSSION

Many viruses, such as EV71, foot-and-mouth disease virus (FMDV), porcine reproductive and respiratory syndrome virus (PRRSV), ZIKV, IAV, as well as some coronaviruses, including transmissible gastroenteritis virus (TGEV), SARS-CoV-2, HCoV-229E, and PEDV, have been demonstrated to induce pyroptosis (21, 25, 26, 35–38, 40, 41, 45–47). Most of these viruses cause pyroptosis mediated by GSDMD, while ZIKV, EV71, FMDV, H7N9 IAV, HCoV-229E, and SARS-CoV-2 have been reported to trigger GSDME-mediated pyroptosis (35–41). As for PDCoV, its impact on pyroptosis remained unknown. Herein, our research reveals that PDCoV infection can induce both GSDMD- and GSDME-mediated pyroptosis *in vitro* and *in vivo*, similar to EV71, IAV, HCoV-229E, and SARS-CoV-2 (25, 35, 39–41, 47). Notably, we found that GSDME dominates the pyroptosis triggered by PDCoV infection (Fig. 3C through F). Our findings provide valuable insight into the mechanisms of virus-induced pyroptosis and the pathogenesis of PDCoV. Additionally, it is noteworthy that GSDMA, another member of the GSDM family, has been reported to be involved in the regulation of pyroptosis during the infection of the African swine fever virus (48). Future investigations are required to determine whether other GSDMs, in addition to GSDMD and GSDME, also contribute to pyroptosis induced by PDCoV infection.

Previous studies have shown that GSDM-mediated pyroptosis serves as an effective defense mechanism against various pathogens, such as viruses (e.g., PEDV and EV71) and bacteria (e.g., *Shigella* and *Streptococcus*) (21–27, 48–51). In this study, we demonstrated that GSDMD can antagonize PDCoV infection by leveraging its pyroptotic capabilities (Fig. 6A through D). Significantly, we observed that PDCoV infection causes a higher fold upregulation of *CASP1* (a pivotal caspase responsible for GSDMD-mediated pyroptosis) and *IL1B* mRNA levels in the duodenum in comparison to the jejunum and ileum (data not show), indicating a more intense GSDMD-mediated pyroptosis in the duodenum. Coincidentally, previous research has demonstrated that viral loads in the duodenum are lower than those in the jejunum and ileum of PDCoV-infected piglets (1, 4, 52, 53), hinting at a potential link between increased GSDMD-mediated pyroptosis and reduced viral load in the duodenum. However, although previous studies have demonstrated that GSDMD knockout downregulates the noncanonical secretion of beta interferon during PDCoV infection, leading to an increase in viral titers, it remains unclear whether GSDMD-mediated pyroptosis is associated with this antiviral mechanism, which requires further exploration.

To evade the suppressive effects of GSDM-mediated pyroptosis on their proliferation, some microorganisms, especially certain viruses, have developed various strategies to disrupt pyroptosis (22, 25, 26, 35, 48, 51, 54–57). Among these strategies, one important mechanism is to impede GSDMD-mediated pyroptosis by cleaving GSDMD through viral proteases, such as Seneca Valley virus, EV71, PEDV, TGEV, and SARS-CoV-2 (21, 25, 26, 35, 56). Our present study also found that PDCoV 3CL^pro^ can cleave GSDMD, but not GSDME, in IPI-2I cells, aligning with previous research (26). Nevertheless, most prior studies have only shown GSDMD cleavage through *in vitro* experiments, not *in vivo*. Consequently, to validate PDCoV infection-induced cleavage, we used the intestinal tissues of pigs infected with PDCoV to demonstrate GSDMD cleavage *in vivo*. As a result, we conclude that GSDMD cleavage by PDCoV infection occurs both *in vitro* and *in vivo*. However, it remains unclear whether PDCoV 3CL^pro^ directly cleaves GSDMD. To investigate this, we utilized AlphaFold3 to predict the structure of GSDMD (Fig. S5A) and constructed a complex structure of PDCoV 3CL^pro^ with GSDMD residues 188–199 (GSDMD_aa188–199_). Molecular dynamics simulations revealed that PDCoV 3CL^pro^ forms multiple stable hydrogen bonds with this short peptide substrate (Fig. S5B), suggesting a potential direct cleavage activity of PDCoV 3CL^pro^ on GSDMD. Nevertheless, we recognize the necessity for further experimental validation to confirm these findings, such as the purification of both PDCoV 3CL^pro^ and GSDMD proteins for *in vitro* cleavage assays.

Our research reveals that porcine GSDME does not exhibit significant resistance to PDCoV proliferation. Similarly, human and mouse GSDMEs have no inhibitory effects on certain microorganisms, such as H7N9 IAV (36, 37, 39). However, GSDME in lower organisms (corals, Pacific oyster *Crassostrea gigas*, and fish) effectively antagonizes the proliferation of microorganisms such as the coral *Pocillopora damicornis* (58–61). Given that GSDME is the most ancient member of GSDMs, we theorized that the varying effects of GSDME from different species on certain pathogens may be related to the evolution of GSDME (62–64). Specifically, the GSDM family of lower animals only contains GSDME and PJVK and no other GSDMs, whereas mammalian GSDMs encompass GSDMA-E and PJVK. Moreover, in addition to GSDME, mammalian GSDMA-D evolved from the ancient ancestor of GSDME. This may imply that in mammals, the ancient ancestor of GSDME, while evolving into different GSDMs, further conferred its functions to different GSDMs, with GSDMD, but not GSDME, gaining antiviral properties. By contrast, lower animal GSDMEs did not undergo this evolutionary differentiation, and thus their function may be closer to that of the ancient ancestor of GSDME, such as the ability to combat microbial infections.

To date, the research on the regulatory roles of GSDMs in inflammatory responses has predominantly concentrated on GSDMD (12, 13, 22, 28, 29, 55). Herein, we also observed the positive impact of GSDMD on PDCoV-induced inflammatory responses. However, we discovered that the smaller fragments resulting from the cleavage of GSDMD by PDCoV 3CL^pro^ were less effective at inducing inflammatory responses compared with GSDMD_N. This suggests the potential involvement of other GSDMs in triggering inflammatory responses when GSDMD is cleaved. In fact, GSDME has been reported to play an important role in promoting inflammatory responses triggered by H7N9 IAV and ZIKV (36, 37). Consistent with these findings, our research, using knockout cell lines and overexpression of GSDME and GSDMD, revealed that GSDME, rather than GSDMD, plays a more critical role in promoting inflammatory responses during PDCoV infection. Interestingly, we found that the small fragments generated from GSDMD cleavage upregulate GSDME expression levels and enhance inflammatory responses. This highlights the importance of investigating the roles of GSDMs beyond GSDMD, especially GSDME, in inducing inflammatory responses by viruses capable of cleaving GSDMD in future studies. Furthermore, we acknowledge that *in vivo* studies using animal models with GSDMD and/or GSDME knockout are important for comparing the roles of different GSDMs in triggering pyroptosis and for exploring their potential complementary effects. Such investigations should be prioritized in future research.

Our study revealed that PDCoV infection can trigger GSDME-mediated pyroptosis through the activation of caspase-3, a mechanism also observed in infections with many other viruses, such as SARS-CoV-2, HCoV-229E, ZIKV, IAV, and EV71 (35–37, 39–41). In addition to caspase-3, several viral proteins, such as FMDV 3CL^pro^ and SARS-CoV-2 nsp3, have been shown to induce GSDME-mediated pyroptosis by cleaving GSDME at the Q271 and G370 positions, respectively (38, 41). However, in our present study, we did not observe GSDME cleavage induced by PDCoV 3CL^pro^, suggesting that PDCoV 3CL^pro^ may not contribute to GSDME-mediated pyroptosis. Furthermore, the potential for nsp3 of PDCoV or other coronaviruses to cleave GSDME and subsequently promote pyroptosis remains unexplored, warranting further investigation.

**Fig 7:**
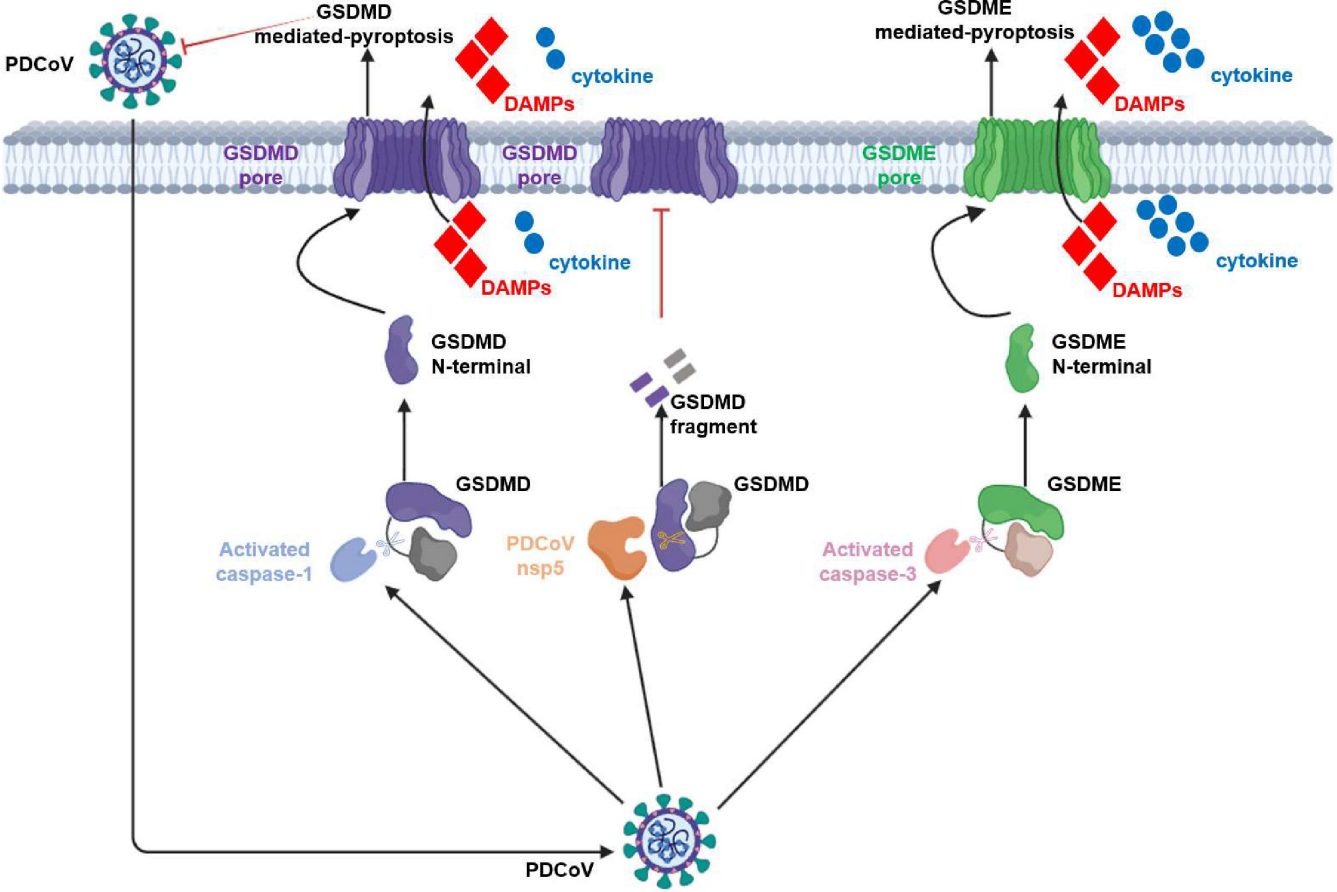
Schematic diagram illustrating the proposed mechanism of PDCoV-induced pyroptosis. PDCoV infection induces pyroptosis through the caspase-1–GSDMD axis and the caspase-3–GSDME axis. Both GSDME- and GSDMD-mediated pyroptosis contribute to the inflammatory responses induced by PDCoV infection, with GSDME-mediated pyroptosis playing a more significant role. Additionally, GSDMD-mediated pyroptosis antagonizes PDCoV proliferation, while PDCoV nsp5 cleaves GSDMD to prevent its antiviral effects.

In conclusion, we report that PDCoV infection induces both GSDMD- and GSDME-mediated pyroptosis, with GSDME playing a prominent role in inducing pyroptosis and subsequent inflammatory responses during PDCoV infection. Moreover, GSDMD exhibits antiviral properties against PDCoV infection through its pyroptotic function, but its anti-PDCoV activity is hindered by viral 3CL^pro^-mediated cleavage. This cleavage also diminishes the pyroptotic and proinflammatory capabilities of GSDMD (Fig. 7). Our study offers new insight into PDCoV pathogenesis and the distinct roles of different GSDMs in pathogenic infections.

## MATERIALS AND METHODS

### Cells and virus

IPI-2I cells (porcine ileum epithelial cells) were obtained from the China Center for Type Culture Collection (China). HEK-293T cells (human embryonic kidney 293 cells) were purchased from the American Type Culture Collection (USA). IPI-2I and HEK-293T cells were cultured in Dulbecco’s modified Eagle’s medium (Gibco, USA). All cells were cultured in medium supplemented with 10% fetal bovine serum (TransGen, China) and incubated at 37°C with 5% CO_2_. The PDCoV strain CHN-HN-2014 (GenBank accession number KT336560) was isolated from a pig with severe diarrhea in China.

### Reagents

PI was purchased from Sigma-Aldrich (USA). Rabbit antibody against GSDMD was purchased from Abmart (China). Rabbit antibodies against β-tubulin and ASC were purchased from Abclonal (China). Rabbit antibody against caspase-3 was purchased from Cell Signaling Technology (USA). Mouse antibodies against Myc and HA were purchased from MBL (Japan). The mouse antibody against PDCoV nucleocapsid (N) protein and the rabbit antibody against porcine GSDME were prepared as described previously (4, 65). The mouse antibody against porcine GSDMD was kindly provided by Dr. Wen-Long Zhang from Northeast Agricultural University (43).

### Plasmid construction

Eukaryotic expression plasmids containing the full-length cDNA of porcine GSDMD and GSDME (GSDMD_FL and GSDME_FL) were constructed by amplifying cDNAs from IPI-2I cells and cloning them into the pCAGGS vector with an N-terminal Myc tag (pCAGGS-Myc). Eukaryotic expression plasmids of GSDMD_N, GSDMD_1–193, GSDMD_194–279, and GSDME_N were constructed by PCR amplification using the GSDMD_FL or GSDME_FL plasmids as templates and subsequently inserted into the pCAGGS-Myc vectors. The glutamine (Q) at position 193 of GSDMD was mutated to alanine (A) by PCR amplification to generate GSDMD-Q193A. The eukaryotic expression plasmids encoding PDCoV nsp5 and its mutants were described previously (66).

### Cell cytotoxicity and viability

Cell death and cell viability were analyzed by detecting the levels of LDH released into cell supernatants and intracellular ATP using the CytoTox 96 Non-Radioactive Cytotoxicity Assay Kit (Promega, USA) and the CellTiter-Glo Luminescent Cell Viability Assay Kit (Promega) according to the manufacturer’s instructions.

### PI staining

IPI-2I cells were infected with PDCoV for 24 h, followed by washing with phosphate-buffered saline. Then, the cells were stained with PI (4 µM) for 10 min at 37°C and visualized using a fluorescence microscope (Olympus, Japan).

### TEM analysis

Samples were fixed with 2% vol/vol glutaraldehyde in 0.05 M sodium phosphate buffer (pH 7.2), postfixed in 2% osmium tetroxide, dehydrated in acetone, and embedded in epoxy resin. Subsequently, staining was performed with a 2% uranium acetate saturated alcohol solution and 2.6% lead citrate before examination using a Hitachi HT7800/HT7700 transmission electron microscope.

### Immunohistochemical analysis

For immunohistochemical analysis, tissue sections were stained with a specific antibody against the PDCoV N protein. The stained sections were examined and photographed using a microscope.

### Caspase-1 activity assay

Caspase-1 activity was measured using the Caspase-1 Activity Assay Kit (Colorimetric) purchased from Abbkine (China). All procedures were performed according to the manufacturer’s instructions.

### Enzyme-linked immunosorbent assay (ELISA)

The secretion of IL-1β in the supernatants was detected using porcine IL-1β ELISA kits purchased from R&D Systems (USA). Additionally, the levels of IL-6 and IL-8 in the supernatants were measured using porcine IL-6 and IL-8 ELISA kits, respectively, purchased from Bioswamp (China). All steps were performed following the manufacturer’s instructions.

### Western blot analysis

Cells were lysed in a pH 6.8 lysis buffer (4% SDS, 3% DTT, 0.065 mM Tris-HCl, and 30% glycerin) supplemented with a protease inhibitor (cocktail) and a phosphatase inhibitor cocktail (Beyotime, China). Equal amounts of proteins were loaded and separated by sodium dodecyl sulfate polyacrylamide gel electrophoresis, and then transferred onto polyvinylidene difluoride membranes. The membranes were blocked in Tris-buffered saline with 0.05% Tween 20 (TBST) containing 5% nonfat milk, followed by incubation with the indicated primary antibodies at 37°C for 3 h. After three washes with TBST, the membranes were incubated with horseradish peroxidase-conjugated secondary antibodies (Beyotime) for 1 h at room temperature. After three washes with TBST, bands on the membranes were visualized using enhanced chemiluminescence reagents (Bio-Rad, USA). The expression of β-Tubulin was used as a loading control.

### RNA extraction and quantitative real-time PCR (qRT-PCR)

Total RNA was extracted using TRIzol reagent (Invitrogen, USA). Subsequently, 1 µg of RNA from each sample was reverse transcribed to cDNA with the cDNA Synthesis Kit (Vazyme, China). All qPCR experiments were performed using SYBR green PCR mix (Vazyme) and an ABI 7500 real-time PCR system (Applied Biosystems). The sequences of the primers are available upon request.

### Generation of knockout cell lines

IPI-2I cells with GSDMD or GSDME gene knockout were constructed using a CRISPR/Cas9 system. Specifically, oligonucleotides designed to yield single-guide RNAs (sgRNAs) targeting exon 2 of the GSDMD (sgRNA: 5′-GAACGTGTGCACGCTACGA-3′) or GSDME (sgRNA: 5′-AGCTCATCAGGGATGCCCAG-3′) gene were annealed and cloned into a *Bbs*I-digested PX459 plasmid. The IPI-2I cells were transiently transfected with these constructs using jetPRIME transfection reagent (Polyplus, France). After 36 h, transfection-positive cells were isolated by adding 2 µg/mL puromycin for 48−72 h. Subsequently, single-cell clones were obtained through limiting dilution assays and identified by sequencing and western blot analysis using specific antibodies against GSDMD or GSDME.

### Median tissue culture infectious dose (TCID_50_) assay

The TCID_50_ assays were performed as described previously (67). In brief, LLC-PK1 cells were seeded in 96-well plates and infected with serial 10-fold dilutions of the viral sample with eight replicates. The plates were incubated for 48–72 h before virus titers were determined. The titers of PDCoV were calculated using the Reed–Muench method and presented as TCID_50_ per milliliter.

### Statistical analysis

GraphPad Prism 8 software was used for data analysis using a two-tailed unpaired *t*-test or one-way ANOVA. All statistical analyses were performed using GraphPad Software (GraphPad Inc., USA).

## Data Availability

All methods and data described in this article are available from the corresponding authors upon request.

## SUPPLEMENTAL MATERIAL

Supplemental figures jvi.01876-24-s0001.docx Figures S1 to S5.

ASM does not own the copyrights to Supplemental Material that may be linked to, or accessed through, an article. The authors have granted ASM a non-exclusive, world-wide license to publish the Supplemental Material files. Please contact the corresponding author directly for reuse.
