# Supplementary material for: GSDMD and GSDME exhibit distinct roles in enteric coronavirus PDCoV-induced pyroptosis and inflammatory responses
Source: J Virol. 2025 Jun 12;99(7):e01876-24. doi: 10.1128/jvi.01876-24 (PMC12282075; doi:10.1128/jvi.01876-24)
Supplement: Supplemental figures — Figures S1 to S5. [file jvi.01876-24-s0001.docx]

**Supplementary Figures**

**Figure S1.** Immunohistochemical analysis of porcine intestinal tissues.

**Figure S2.** PDCoV infection upregulates mRNA levels of proinflammatory cytokines.

**Figure S3.** Nsp5-mediated cleavage of GSDMD reduces the mRNA levels of proinflammatory cytokines.

**Figure S4.** Knockout of GSDMD, but not GSDME, enhances the proliferation of PDCoV.

**Figure S5.** Molecular dynamics simulations of PDCoV 3CL^pro^ in complex with the substrate GSDMD_aa188-199_.


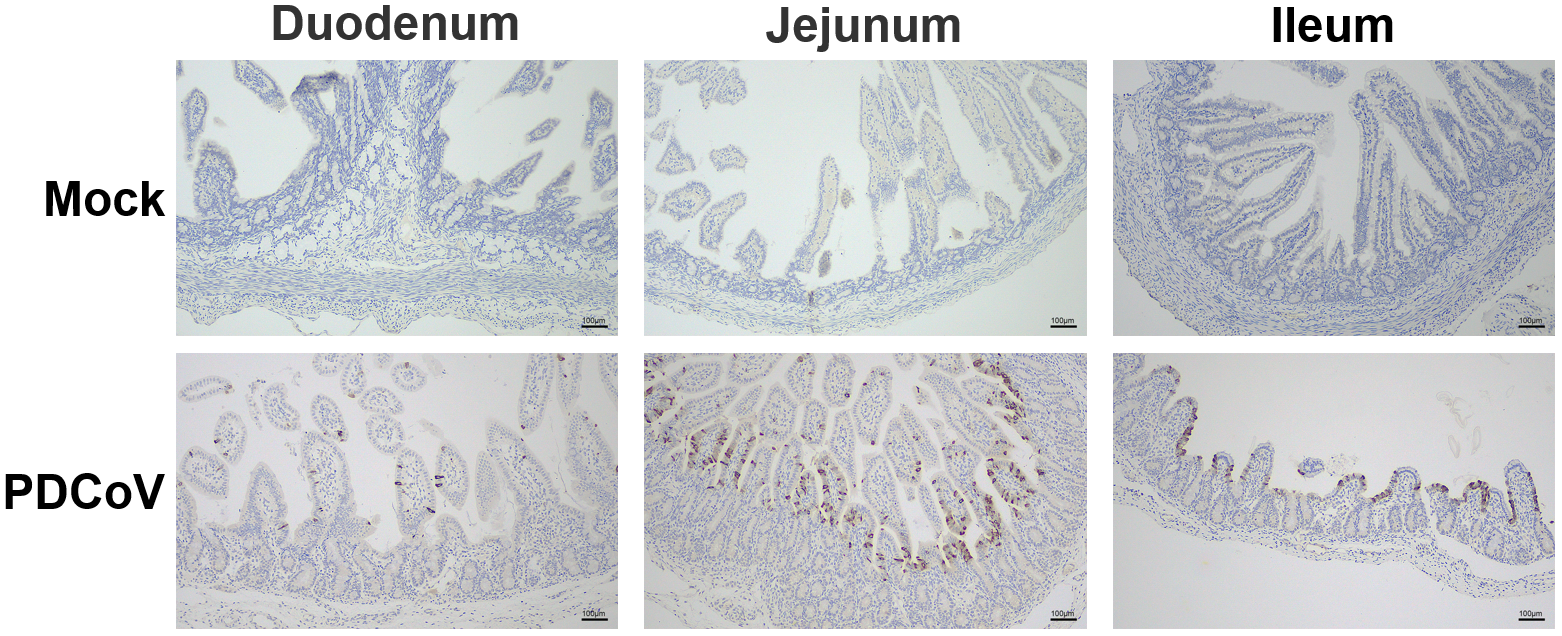
**Figure S1**

**Fig. S1.** **Immunohistochemical analysis of porcine intestinal tissues.** Tissue sections of the duodenum, jejunum, and ileum tissues from PDCoV-infected and mock-infected piglets were stained using a specific antibody against the viral N protein. Scale bar = 100 µm.

**Figure S2**


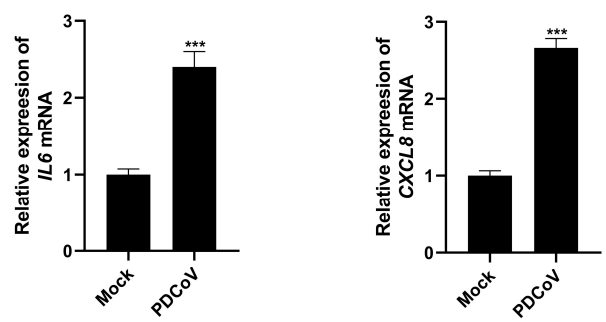


**Fig. S2.** **PDCoV infection upregulates mRNA levels of proinflammatory cytokines.** IPI-2I cells were infected or mock-infected with PDCoV (0.5 MOI) for 24 h. The relative mRNA levels of *IL6* and *CXCL8* were analyzed by qRT-PCR. *ACTB* was used as the internal control. Values are shown as the mean ± SD from three independent experiments. ***, p < 0.001.

**Figure S3**


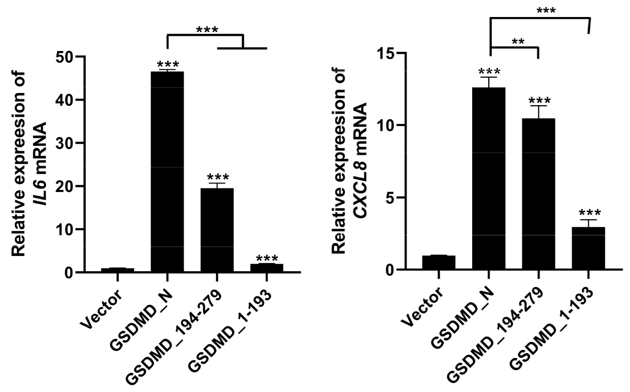


**Fig. S3.** **Nsp5-mediated cleavage of GSDMD reduces the mRNA levels of proinflammatory cytokines.** IPI-2I cells were transfected with plasmids encoding GSDMD_N, GSDMD_1-193, and GSDMD_194-279. Cell samples were collected at 24 h after transfection and the relative mRNA levels of *IL6* and *CXCL8* were analyzed by qRT-PCR. *ACTB* was used as the internal control. Values are shown as the mean ± SD from three independent experiments. **, p < 0.01; ***, p < 0.001.


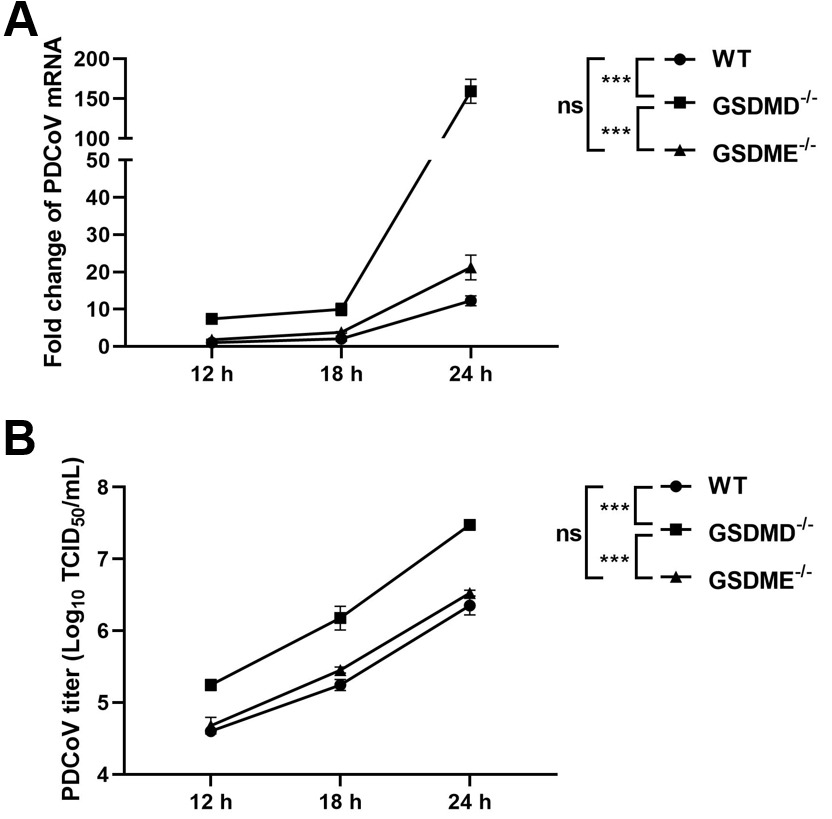
**Figure S4**

**Fig. S4.** **Knockout of GSDMD, but not GSDME, enhances the proliferation of PDCoV.** WT, GSDMD^-/-^, and GSDME^-/-^ IPI-2I cells were infected with PDCoV at an MOI of 0.5 for 12, 18, and 24 h. The PDCoV RNA copy number (A) and viral titers (B) were measured. Values are shown as the mean ± SD from three independent experiments. ***, *p*<0.001; ns, not significant.

**Figure S5**


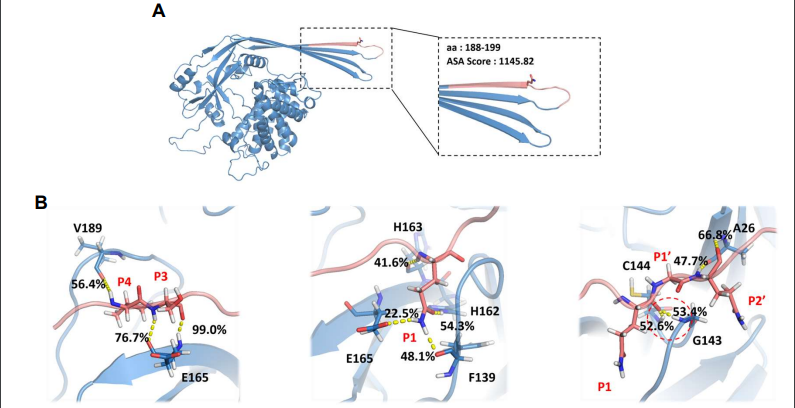


**Fig. S5. Molecular dynamics simulations of PDCoV 3CL^pro^ in complex with the substrate GSDMD_aa188-199_. (A)** A schematic representation of the predicted structure of GSDMD. The accessible surface area scores for GSDMD_aa188-199_ were calculated using PSAIA. **(B)** Representation of the hydrogen bonding network and occupancy in the active site of PDCoV 3CL^pro^ in complex with the GSDMD_aa188-199_ substrate. The red dashed circle represents the conserved oxyanion hole, which is critical for substrate cleavage. Hydrogen bond interactions are shown as yellow dashed lines, with the accompanying percentage indicating hydrogen bond occupancy throughout the 200 ns molecular dynamics simulations.
